# CHOICE-AYA: adapting an evidence-based contraceptive counseling intervention for adolescents and young adults experiencing homelessness

**DOI:** 10.3389/frph.2026.1787996

**Published:** 2026-04-10

**Authors:** Mariah E. Brewe, Anna E. Burns, Nik Schuetz, Taylor Stephens, Emma Francis, Haylie Yost, Stephani L. Stancil

**Affiliations:** 1 Division of Adolescent and Young Adult Medicine, Children’s Mercy, Kansas City, MO, United States; 2 Division of Clinical Pharmacology, Toxicology and Therapeutic Innovation, Children’s Mercy, Kansas City, MO, United States; 3 Division of Health Services and Outcomes Research, Children’s Mercy, Kansas City, MO, United States; 4 University of Kansas School of Medicine, Kansas City, KS, United States; 5 Department of Pediatrics, University of Missouri, Kansas City, MO, United States

**Keywords:** adolescents, contraception, homelessness, patient-centered communication, unhoused, young adults

## Abstract

**Introduction:**

Adolescents and young adults (AYA) experiencing homelessness face an increased risk of reproductive violence, unintended pregnancy, and barriers accessing healthcare. While interventions focused on efficacy-first contraceptive counseling are successful at increasing rates of long-acting reversible contraceptive (LARC) use, little is known about how an efficacy-first approach is received by AYA experiencing homelessness given unique factors associated with their reproductive health care. We aimed to adapt the evidence-based CHOICE contraceptive counseling approach for AYA experiencing homelessness.

**Methods:**

Focus groups discussion with AYA experiencing homelessness were conducted to understand prior reproductive health care experiences and contraception counseling preferences, comparing efficacy first and patient-centered approaches and counseling scripts. De-identified self-reported demographics and prior reproductive health history were collected.

**Results:**

Four focus groups were conducted with 41 AYA experiencing homelessness (age 12–23 years old). AYA preferred a patient-centered counseling approach and adapted a contraceptive counseling script accordingly. AYA's additional suggestions to improve contraceptive counseling were synthesized into three themes: (1) How counseling should be delivered, (2) What counseling should include, and (3) Where counseling should occur.

**Discussion:**

AYA preferred patient-centered counseling with critical nuance relevant to their lived experiences. The adapted CHOICE-AYA contraceptive counseling script will assist providers in meeting the needs and desires of AYA experiencing homelessness. Further suggestions to optimize counseling focus on individualized, confidential consultations for all patients regardless of pregnancy potential and extension of resources to community and non-traditional healthcare settings (e.g., emergency departments). Current reproductive health guidelines can be expanded to include these considerations relevant to AYA experiencing homelessness.

## Introduction

Over 4.2 million adolescents and young adults (AYA) experience homelessness in the United States each year. Those parenting, lacking a high school diploma, involved in foster care, or of sexual, gender, or racial minority are disproportionately affected (1, 2). AYA experiencing homelessness have poor access to reproductive health care, increased risk of reproductive violence, and higher rates of unintended pregnancy compared with stably housed peers (3–5). Unintended pregnancy in adolescence is associated with low educational attainment, reduced earning capacity, and morbidity in the parent and child that contribute to increased health care expenditures (2, 6). Same-day access to highly effective contraception, e.g., long-acting reversible contraception (LARCs) for AYA leads to a three-fold reduction in unintended pregnancy and elective abortion (7–9), yet cost and patient/provider knowledge deficits regarding LARCs continue to be significant barriers to LARC use (5). Efficacy-first contraceptive counseling interventions that lead with the discussion of LARCs (e.g., the contraceptive CHOICE project) have been effective in combating some of these barriers. When coupled with same-day access to contraception, the CHOICE project demonstrated high rates of LARC selection, continuation, and satisfaction (8). Despite these findings, efficacy-first contraceptive counseling has been critiqued as potentially coercive by interfering with a patient's reproductive autonomy (10, 11).

Alternatively, the current CDC recommended standard of care is a patient-centered approach—counseling structured to elicit the goals and preferences of the patient and promote shared decision making between patient and provider (12). Descriptive reports from The Right Time project, an initiative aimed to increase contraceptive access at health centers across Missouri, showed increased use of hormonal contraception (e.g., oral contraceptive pills, injection, intrauterine device) and decreased reliance on condoms following care that was consistent with a patient-centered approach (13). When patient-centered contraceptive counseling needs are met, patients report high rates of satisfaction with counseling and high rates of receiving their preferred contraceptive method (13). By assessing patient preferences and contextual factors prior to the presentation of contraceptive methods, patient-centered contraceptive counseling holds the potential to better accommodate a wide range of patients' needs. AYA experiencing homelessness may benefit from this approach as additional contextual concerns (e.g., reproductive coercion, engaging in sex for safety or a place to stay) and logistical challenges (e.g., lack of reliable transportation, being uninsured or underinsured) can be addressed at the forefront.

Additionally, psychological barriers to seeking contraceptive care faced by AYA experiencing homelessness (e.g., perceived health care stigmatization, provider bias, fear of judgement, and concerns of confidentiality) necessitate an approach aimed at creating a positive healthcare interaction (14). Patient-centered approaches feature high interpersonal quality and shared decision making that boost adult and AYA satisfaction with care (13, 15, 16), yet evidence is limited regarding their application with AYA experiencing homelessness. Further, the lack of knowledge regarding reproductive health care preferences of AYA experiencing homelessness paired with the potential impact of their distinct experiences and challenges highlights a critical gap in evidence-based care. Thus, this study aimed to adapt the evidence-based CHOICE contraceptive counseling approach for AYA experiencing homelessness to promote positive patient-provider interactions and informed decision making.

## Materials and methods

We conducted a mixed methods study to adapt the evidenced based CHOICE contraceptive counseling intervention for AYA experiencing homelessness through a Patient-Centered Communication framework (8, 12). This study was approved by the Institutional Review Board at Children's Mercy Kansas City. Verbal consent was obtained from all participants. Based on Missouri state law that allows minors to consent to reproductive health care, a waiver of parent/guardian permission/consent was granted to be consistent with clinical care and protect confidentiality. A waiver of documentation of consent was obtained to mitigate the risk of loss of confidentially as no other protected health information (PHI) was collected during the study. The study was also reviewed and approved by the Missouri Department of Social Services Privacy Board to allow recruitment of wards of the state who may be staying in emergency shelter or transitional housing.

### Setting and participants

English-speaking AYA currently staying in an emergency youth shelter (aged 12–18 years) or independent transitional housing (aged 16–24 years) in a Midwest metropolitan area were eligible for enrollment if they indicated a history of prior sexual activity or anticipated future sexual activity that creates the risk for pregnancy. Following review of the consent form and eligibility criteria, verbal consent was obtained, and eligibility was implied if AYA proceeded to the FGD. All participants received a $25 gift card. All participants were made aware verbally and in writing that their participation would not affect any medical care they receive through the existing partnership between the academic medical center and the community organization.

### Data collection and approach

Participants completed paper surveys for self-reported demographics, awareness and prior use of contraception (see Supplementary Material S1 for Focus Group Participant Survey) and participated in a semi-structured focus group discussion moderated by principal investigator and board-certified clinician specializing in adolescent and young adult medicine (SS). FGDs were selected over individual interviews as they allowed for recruitment through standard group programming at the community organization, an environment familiar to participants, and supported consensus building among the group. To reduce social desirability bias during discussion, the moderator started with an introduction of ground rules and emphasized that there were no right or wrong answers, to be respectful of the opinions of other participants, and to not discuss the responses of other participants outside of the FGD. FGDs lasted approximately 1 h. FGDs included an exploration of prior experiences with contraceptive counseling and education, presentation of the efficacy-first CHOICE script published by Secura et al (8) and clinically derived patient-centered counseling script via role-play, selection by the group of their preferred script and further suggestions to improve contraceptive counseling. Iterative script revisions based on feedback from each FGD led to the final CHOICE-AYA contraceptive counseling script. The initial FGD guide was developed pragmatically and followed a funneling technique where topics started broad, then narrowed in on specific details. Subsequent FGD guides involved member checking from prior groups and additional funneling for specifics (17) (see Supplementary Material S2 for collated Focus Group Discussion Guide). Members of the research team present at FGDs agreed that thematic saturation was reached following the fourth FGD. If AYA had specific health-related questions following focus group participation, an appointment with the onsite medical clinic was facilitated.

### Data analysis

Data analysis was informed by the Patient Centered Communication (PCC) framework including the four-part operational definition from Epstein et al. (18): Eliciting and understanding the patient's perspective; understanding the patient within their unique context; reaching a shared understanding of the problem and treatment that aligns with patient values; and shared power and responsibility that involves patients in making decisions. Focus group transcripts were analyzed in Dedoose (v9.2.12) using a hybrid inductive/deductive coding approach. Line-by-line coding, led by a PhD research associate with experience in qualitative analysis (NS) with oversight by the principal investigator (SS) and assisted by post-baccalaureate and master's level team members (AB, MB), included deductive codes from the PCC framework and focus group guides and inductive codes that emerged from the data. Each transcript was coded independently, and coders met regularly to review coding strategies, resolve discrepancies, and come to a consensus on the codebook. Code summaries were created to further synthesize the data, then reflexive thematic analysis was used to identify common themes (19). Data triangulation enhanced the trustworthiness and credibility of this study by analyzing data from two separate programs: emergency shelter and transitional housing (20, 21). Initially, data from each focus group was evaluated independently and then collated for final analysis given similarity of themes across groups. Representative quotes are provided in Results. Demographics were self-reported in aggregate to protect confidentiality; thus, individual demographics attributable to each quotation are not available.

### Reflexivity statement

All team members share the perspective that access to comprehensive reproductive health care, including family planning and sexual health, is an important right to be extended to all patients. Several members of the team, including the PI (SS) who moderated FGDs, have professional and clinical experience providing health services to unhoused individuals in the US. These experiences and values are important to acknowledge for better understanding the lens through which the data were collected and interpreted.

## Results

### Participant characteristics

Four mixed-gender focus groups were conducted with 41 AYA experiencing homelessness (Table 1). Two focus groups occurred with AYA staying in an emergency shelter and two with AYA in transitional housing.

**Table 1:** Participant demographics.

Participant demographics	
Age, mean ± SD (range), years^a^	18 ± 2.4 (12–23)
Race and Ethnicity^a^^,^^b^, *n* (%)	
Asian	1 (2.6)
Black or African American	19 (49)
Native Hawaiian or Other Pacific Islander	1 (2.6)
White	12 (31)
Mixed race	3 (7.7)
Other	3 (7.7)
Hispanic/Latinx	6 (15)
Current Grade or Level of Education^c^, *n* (%)	
7th	1 (2.6)
8th	2 (5.1)
9th	0 (0)
10th	3 (7.7)
11th	5 (13)
12th	9 (23)
Completed high school	7 (18)
Did not complete high school	2 (5.3)
Not currently enrolled in school	6 (16)
Some college	3 (7.7)

^a^ *n* = 39, 2 AYA did not complete.

^b^ AYA were encouraged to select all that applied.

^c^ *n* = 38, 3 AYA did not complete.

### Awareness and prior use of contraception

Some AYA (*n* = 12, 31%) reported no prior use of contraception, and two AYA (*n* = 2, age 12 and 20) reported no contraceptive awareness. Awareness was greatest for the oral contraceptive pill and condom (Table 2). Nearly 1/3 of the group reported prior use of withdrawal, condom, or injection (Table 2). Of those who reported prior use of contraception (*n* = 27, 69%), ease of use and availability was most frequently cited as influencing their chosen method (*n* = 7, 26%). Nearly half reported making the decision independently (*n* = 13, 48%), while others reported input from their provider(s) (*n* = 8, 30%), parents/guardians (*n* = 6, 22%), and/or partners (*n* = 5, 19%).

**Table 2:** Awareness and prior use of contraception.

Method^a^	Awareness^b^, *n* (%)	Prior Use^b^, *n* (%)
Pill	35 (90)	9 (23)
Condom	33 (85)	14 (36)
IUD	30 (77)	4 (10)
Internal condom	29 (74)	5 (13)
Implant	28 (72)	6 (15)
Withdrawal	25 (64)	15 (38)
Injection	25 (64)	12 (31)
Not right now^a^	20 (51)	6 (15)
Patch	19 (49)	0 (0)
Sterilization	17 (44)	0 (0)
Ring	13 (33)	1 (2.6)
Emergency contraception	12 (31)	5 (13)
Cervical cup	10 (26)	0 (0)
Fertility awareness	9 (23)	2 (5.1)
Spermicide	6 (15)	0 (0)
Diaphragm	3 (7.7)	0 (0)
Sponge	2 (5.1)	0 (0)

^a^ Methods are listed as they appeared on the visual list from Bedsider.org that was presented to participants on paper surveys; method choice “Not right now” refers to abstinence. Please see Supplementary Material S1 for visual list and survey details.

^b^ *N* = 39, 2 AYA did not answer.

### Adapted CHOICE-AYA contraceptive counseling approach

**Figure 1:**
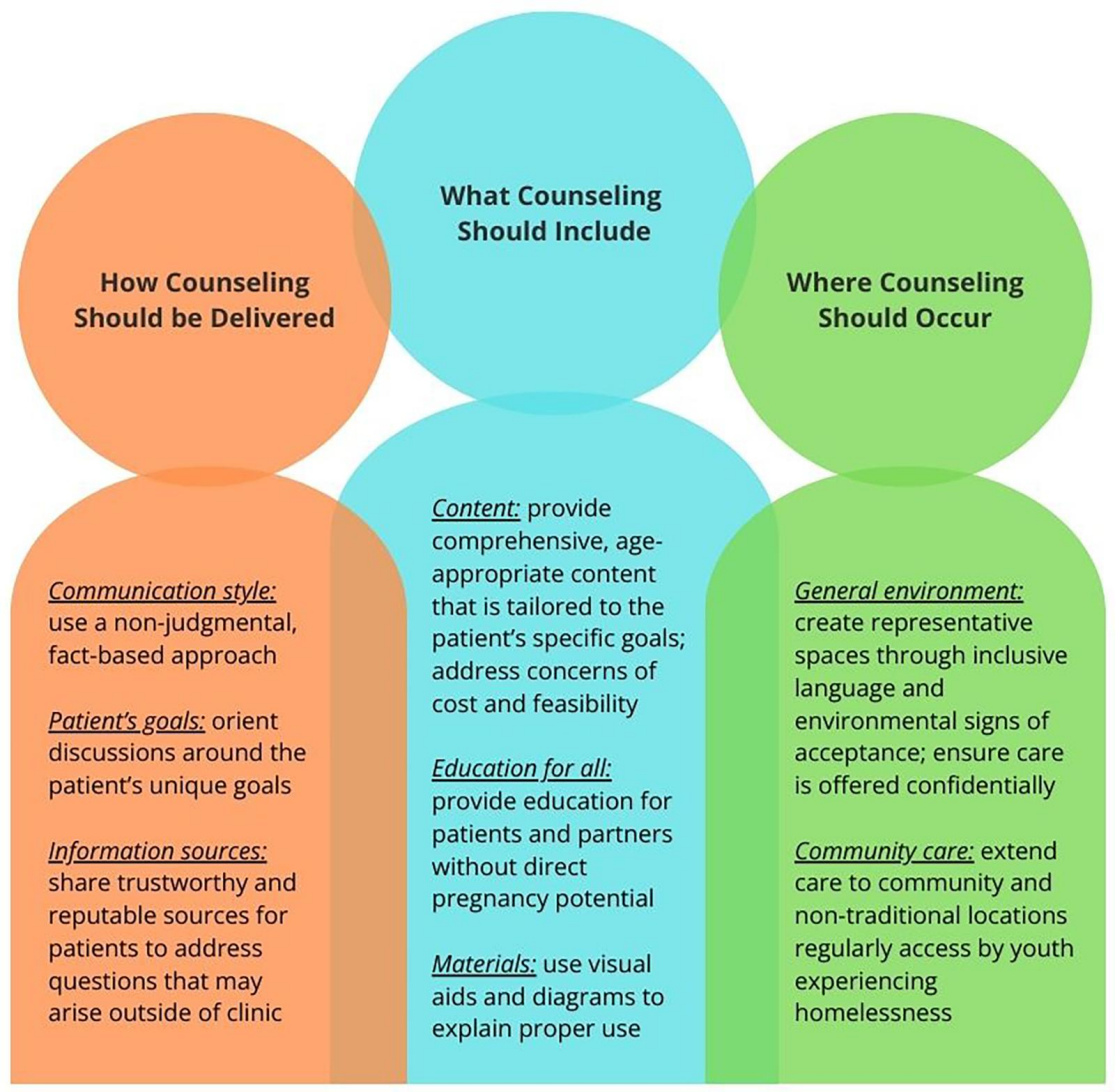
Schematic of AYA feedback for contraceptive counseling. Three overarching themes and subthemes to promote positive sexual and reproductive healthcare interactions for AYA experiencing homelessness.

The final CHOICE-AYA contraceptive counseling script was adapted with feedback from AYA experiencing homelessness and follows a patient-centered counseling approach (see Supplementary Material S3 for CHOICE-AYA Example Script). Upon presentation of efficacy-first (8) and patient-centered counseling scripts, AYA overwhelmingly preferred the patient-centered approach, citing how it gathers the patient priorities for a contraceptive method first, then tailors the order of method presentation and depth of information accordingly. To augment the preferred patient-centered script, AYA shared insights to optimize the reproductive and sexual healthcare experience. We organized these patient-derived insights into 3 categories (Figure 1): (1) How counseling should be delivered, (2) What counseling should include, (3) Where counseling should occur.

### How counseling should be delivered

#### Communication style

AYA appreciated judgment-free, personalized, one-on-one conversations with providers that included fact-based communication, active listening, talking through myths, and thoroughly explaining processes. Some valued provider similarities in race and/or gender, and all valued a conversation unbiased by provider religious or cultural beliefs. Past experiences that negatively impacted their sexual and reproductive healthcare included feeling judged for pregnancy and contraceptive choices, feeling disconnected from their provider, and feeling awkward or lacking the knowledge to know how to ask for contraception.

“I feel like the personalization like piece of the conversation is ideal for an individual conversation between you and an OB, or you and your doctor, your primary care physician.”

#### Patient goals

AYA value a discussion that reflects the wide range of priorities for seeking hormonal contraception (e.g., acne control, period regulation, pregnancy prevention). Regarding family planning, AYA desired increased reproductive autonomy, including access to non-reversable options (e.g., sterilization) and support for those seeking pregnancy. AYA acknowledged that priorities and goals shift over time and highlighted the importance of repeated discussions.

“Having an option or a range of options available to you so you can make the healthiest choice for you and meet your goals, Right? Whatever you wanna do, that’s kinda the whole point.”

#### Information sources

Health care providers were identified as a valued information source. AYA appreciated hearing about the provider's prior experience with patients similar to them (e.g., sharing experiences with contraception for patients that shared the same background). While social media was commonly accessed for sexual and reproductive health information, AYA noted that it is not always reliable and expressed concern that their peers may not be as discerning. Despite this concern, AYA felt social media can provide a starting place to generate sexual and reproductive healthcare questions to discuss with their providers. Beyond discussions with providers, AYA valued having trustworthy and easily accessible resources (e.g., reputable websites) to address misinformation as questions arise.

“The Internet too. That'd be a good place to start… Like having, like, a page dedicated for that. Like a go-to. Or just like, ‘this is legit’.”

### What counseling should include

#### Content

In addition to method efficacy, AYA appreciated comprehensive reproductive content (e.g., discussing reproductive anatomy, family planning, hormonal and non-hormonal options, benefits and side effects, sexually transmitted infections) to support informed decision making. AYA preferred a sex positive discussion that covers communicating consent and boundaries (e.g., safe words). AYA expressed that conversations should be tailored and relevant to patients of all sexualities and gender identities, while they emphasized the importance of providers to “not hold anything back”. Regarding contraceptive counseling specifically, AYA reported feeling less overwhelmed when providers started broad (i.e., listed all options that addressed the primary concern/priority), gathered feedback, then increased the level of detail for methods that continued to meet the patient's identified needs. As part of method detail, information about accessibility such as cost and additional requirements (e.g., regular transportation to pharmacy or clinic) that can impact feasibility should be included. AYA also advocated for additional inclusivity in who reproductive health conversations are had with, desiring that such discussions occur with patients of all sexes and genders regardless of their potential for pregnancy.

“I think when my doctor first talked to me about birth control, she gave me like all the options, which ones are more effective? Which ones are long [term], short [term] …which was very helpful because it helped me narrow down which one I wanted to be on.”

#### Education for males

AYA desired comprehensive contraceptive counseling for male patients and partners. Some male participants had never had conversations about contraception in a healthcare setting, and most expressed interest in receiving education. AYA highlighted several opportunities to provide education for males—one-on-one with providers or jointly through couple's appointments. AYA described couple's appointments as a time for partners without pregnancy potential to receive information about contraception and understand what is involved in the decision-making process.

“I think there is a lot of pressure and responsibility put on the females for the birth control and to be ready and protect yourself… But I think the conversation needs to be equally had with the boys and the men of ‘This is also how you protect yourself and how you protect your partners.’ And talk about vasectomies, all of it.”

#### Materials

AYA valued the use of visual aids (e.g., models, contraceptive samples for demonstration) during contraceptive counseling. Aids often helped AYA make connections between their anatomy and a method's mechanism of action, as one teen expressed desire to visualize “what it has” or “where it would go”. Visual aids used during counseling allowed AYA to better understand contraception's proper use and how it relates to their reproductive health.

“I think visual aids are important, especially when my doctor was telling me about birth control when she had brought up the implant and she was like, oh, it's a rod going in your arm, but like I feel like visual can like really show you what it looks like.”

### Where counseling should occur

#### General environment

AYA experiencing homelessness expressed the importance of accessible, inclusive, and confidential care offered discretely. AYA found comfort when care was inclusive and representative of their identity. This often occurred naturally with providers of the same race and gender, however AYA expressed that provider word choice and materials reflective of diverse patients can promote comfort when these commonalities do not already exist. One participant explained that although her provider is a different race, she has stayed with her for a long time because “she did her homework on me,” and indicated that her provider was knowledgeable about specifics related to women of her race. LGBTQ + AYA found comfort and acceptance through representative symbols in the environment (e.g., pride flags, pronoun pins).

“The Allies flag, any of those stickers would make me comfortable talking to them…and that may be for a lot of people to let them know, okay…I’m accepted here.”

#### Community care

AYA expressed a desire for sexual and reproductive healthcare to exist outside of the traditional clinical environment. They suggested using trustworthy community locations they often accessed (e.g., schools, libraries, businesses designated as Safe Places through nationalsafeplace.org) to house these additional resources. Community outlets were desired for their convenience in accessing care for time-sensitive needs (e.g., emergency contraception) and for the discretion that receiving support outside of the healthcare system can provide, especially if there are domestic concerns (e.g., reproductive coercion, parent/guardian judgement, or lack of family support).

“A lot of people would be more comfortable going by a Safe Place, getting that [emergency contraception] for themselves…a lot of people get scared or embarrassed of their parents or religion…[it] would be a whole lot easier just to be responsible and handle that privately.”

## Discussion

AYA showed a preference for a patient-centered approach to contraceptive counseling, affirming current recommended practices (12, 22). AYA experiencing homelessness disproportionately experience negative healthcare interactions that lead to a reluctance to seek future care and poor health outcomes, making patient-informed additions to contraceptive counseling critical to break this cycle. AYA in our study guided the development of the patient-centered CHOICE-AYA contraceptive counseling script and provided insight into the “how”, “what”, and “where” of optimal reproductive healthcare to facilitate its implementation. Our findings of the “how”, “what”, and “where” align well with the PCC framework and are discussed below within that context.

Regarding how care should be delivered and what it should include, AYA highly valued identifying their unique goals and tailoring contraceptive counseling accordingly. This desire parallels the PCC principles of eliciting the patient's perspective and reaching a shared understanding of the problem. AYA experiencing homelessness also valued a discussion of potential logistical barriers to contraceptive access and feasible solutions. The suggestion for providers to assist in problem-solving supports the PCC consideration of the patient's unique psychosocial context and how it influences their care needs. For example, if youth lack reliable transportation, refilling a monthly prescription at the pharmacy (e.g., pill, patch, ring) may not be sustainable. Some pharmacies provide delivery services to shelters and community organizations which can mitigate this issue for some. Same day access to the long-acting reversible methods like implant and IUD can remove certain logistical issues (e.g., regular transportation to pharmacy, funds to cover prescription cost) and provide highly effective contraception. As providers play a key role in balancing contraceptive preferences with time-sensitive needs and logistical concerns, a patient-centered approach is not meant to bar early presentation of LARCs or efficacy-first counseling. Rather, the identification of patient priorities and limitations allow for a strategic presentation of methods that meet patient's unique needs, which may or may not involve presenting the most efficacious methods first.

Additional “how” and “what” insights from AYA address comprehensive education within clinic, which youth would like to see shared with all patients regardless of their potential for pregnancy. Broader conversations of reproductive health can improve knowledge and create an open space for patients to seek support for themselves and their partner when ready. Of note, language used by AYA (i.e., “Education for Males”) was reflected in our results but can be extended to all patients without direct pregnancy potential (e.g., patients in same-sex relationships). AYA reported positive experiences when past providers utilized visual aids. Images and hands-on models aided in their understanding of the proper use of each method and assisted in identifying which method best aligned with their values. Freely available materials that are representative of a diverse group of patients can be leveraged as visual aids (e.g., https://Bedsider.org). Relatedly, AYA requested discussion around reputable sites they could access for trustworthy reproductive healthcare information when they are outside the healthcare setting (e.g., adolescenthealth.org provides downloadable resource sheet here, https://reproductiveaccess.org).

Considering “where” contraceptive counseling should occur, AYA appreciated environmental signals of acceptance (e.g., pride flags, pronoun pins) and highly valued one-on-one provider time. The desire for one-on-one discussions is consistent with research showing that confidential time is associated with adolescents having better experience and carrying a positive attitude toward healthcare (23, 24). Clinicians may perceive time constraints as a barrier to confidential discussions during the visit (25), yet positive perceptions from patients and inclusion in expert guidance (e.g., American Academy of Pediatrics, Society for Adolescent Health and Medicine, American College of Obstetrics and Gynecologists) for all pediatricians emphasize the importance of creating space for one-on-one patient and provider interactions (22, 26, 27).

Another notable element of “where”, AYA in our study suggested increasing access to contraceptive care in non-traditional settings. Schools, community centers, and even local Safe Place businesses (certified by National Safe Place Network) were mentioned as settings that would be convenient to access reliable information, time sensitive care (e.g., emergency contraception), and referral for more extensive needs. Prior work has shown that interventions focused on delivering educational resources for sexual and reproductive health within the school setting strengthen positive attitudes towards reproductive health (28). The desire for reproductive healthcare within shelter locations has previously been reported (29), and implementation of a pilot program within a youth shelter has shown success. Following intake and a brief screening for individual needs, over three-fourths of youth at an emergency shelter accepted recommended reproductive healthcare (e.g., condoms, emergency contraception, pregnancy testing, referral for a future clinic visit) (30). AYA desires for greater contraceptive care access within the community is consistent with SAHM's call for nonclinical professionals who work with AYA (e.g., educators, community-based program staff) to serve as a resource for sexual and reproductive health and rights (22). Future interventions can strengthen collaboration between healthcare systems and community locations to support timely access to contraception, education, and referral. It will also be necessary to develop training models to support nonclinical professionals in this serving in this role.

Further, emergency departments (ED) can be leveraged for reproductive health services, particularly because they are more likely to be utilized by AYA experiencing homelessness for routine healthcare compared with their stably housed peers. Contraceptive counseling interventions implemented in the ED have shown high rates of intention to initiate contraception (31). As digital and online resources are specifically desirable by AYA (29), Miller et al. adapted a computerized decision support tool for use in the ED. With use of this tool, patients were more likely to receive reproductive healthcare (e.g., same-day oral contraception, condoms, STD testing and treatment, referral for follow-up care) compared to controls who received only paper and print resources (32). Whether facilitated through non-traditional settings or online resources, positive healthcare experiences and broader access is essential for AYA as it sets a foundation for good healthcare consumerism in the future. The CHOICE-AYA script and patient-derived supplemental insights can be used directly to improve the reproductive health care of AYA experiencing homelessness.

Our findings may inform special considerations in public health policy for AYA experiencing homelessness. These include access to confidential, comprehensive reproductive health care that includes inclusive counseling, same-day options, and STI testing in non-traditional settings (e.g., community centers, schools, safe harbors) in addition to traditional settings (e.g., hospitals and clinics). A relatively low resource, first step can be increasing access to emergency contraception in non-traditional settings as requested by AYA experiencing homelessness. Provision of emergency contraception can be paired with connections to care through partnerships with community organizations already serving youth and healthcare groups.

### Strengths and limitations

Our study was designed to include insights from a wide age range of AYA experiencing homelessness in various settings (e.g., emergency shelter, transitional living program). Our sample included mixed gender AYA and was not contingent on prior sexual activity, allowing us to elicit feedback from patients with prior experience seeking contraceptive healthcare and direct pregnancy potential, as well as suggestions from AYA who have not received sexual and reproductive healthcare but desired such future care. Self-reported contraception awareness and prior use was collected to descriptively characterize our study population and facilitate appropriate contextualization of our findings. Identifying a specific effect of prior contraceptive use, awareness, or prior sexual experiences on contraceptive counseling needs is outside the scope of this study.

We were unable to evaluate knowledge and prior use of contraception by gender or sex at birth as this was not reported on paper surveys. Reported prior use of condoms and oral contraception in our sample was consistent with use in other samples of mixed gender AYA as reported by the CDC's Youth Risk Behavior Survey (33). Self-reported race in our sample was consistent with recent demographic report of sheltered youth experiencing homelessness in our region and supports the regional representativeness of our findings (34). Our study was conducted with AYA who were connected to shelter or transitional housing services. The adapted CHOICE-AYA approach likely has the greatest potential to benefit providers and youth within similar service settings.

## Conclusion

AYA experiencing homelessness valued a patient-centered approach to contraceptive counseling that centers on individual goals, barriers, and unique circumstances. These preferences guided the development of the patient-centered CHOICE-AYA counseling script and offered additional insights into optimal contraceptive care for this specialized group. The adapted CHOICE-AYA counseling script along with specific recommendations (e.g., use of visual aids, inclusive environments, representative materials, and extension of care to non-traditional settings) are evidence-based, aligned with national guidance (12), and support AYA experiencing homelessness in making informed reproductive care decisions. Future work may evaluate the impact of this adapted CHOICE-AYA approach on sexual and reproductive health empowerment in addition to contraceptive uptake, satisfaction, and continuation.

## Data Availability

Data may be made available upon reasonable request to the corresponding author following relevant regulatory approval(s).
