# Supplementary material for: CHOICE-AYA: adapting an evidence-based contraceptive counseling intervention for adolescents and young adults experiencing homelessness
Source: Front Reprod Health. 2026 Apr 10;8:1787996. doi: 10.3389/frph.2026.1787996 (PMC13106319; doi:10.3389/frph.2026.1787996)
Supplement: Supplementary file 1 [file Table1.docx]

**Focus Group Survey**

We want your feedback! Your answers are totally PRIVATE. Please answer these questions as honestly as you can. Thank you!

1. How old are you? ___________
2. What grade are you in?
3. 8^th^ grade
4. 9^th^ grade
5. 10^th^ grade
6. 11^th^ grade
7. 12^th^ grade
8. Other (Specify): ______________________
9. What is your race?
10. White
11. Black or African American
12. American Indian or Alaska Native
13. Asian
14. Native Hawaiian or Other Pacific Islander
15. Other (Specify):  ______________________
16. Are you Hispanic/Latino?
17. Yes
18. No
19. What options/methods have you heard of for preventing pregnancy? [Circle all that apply]


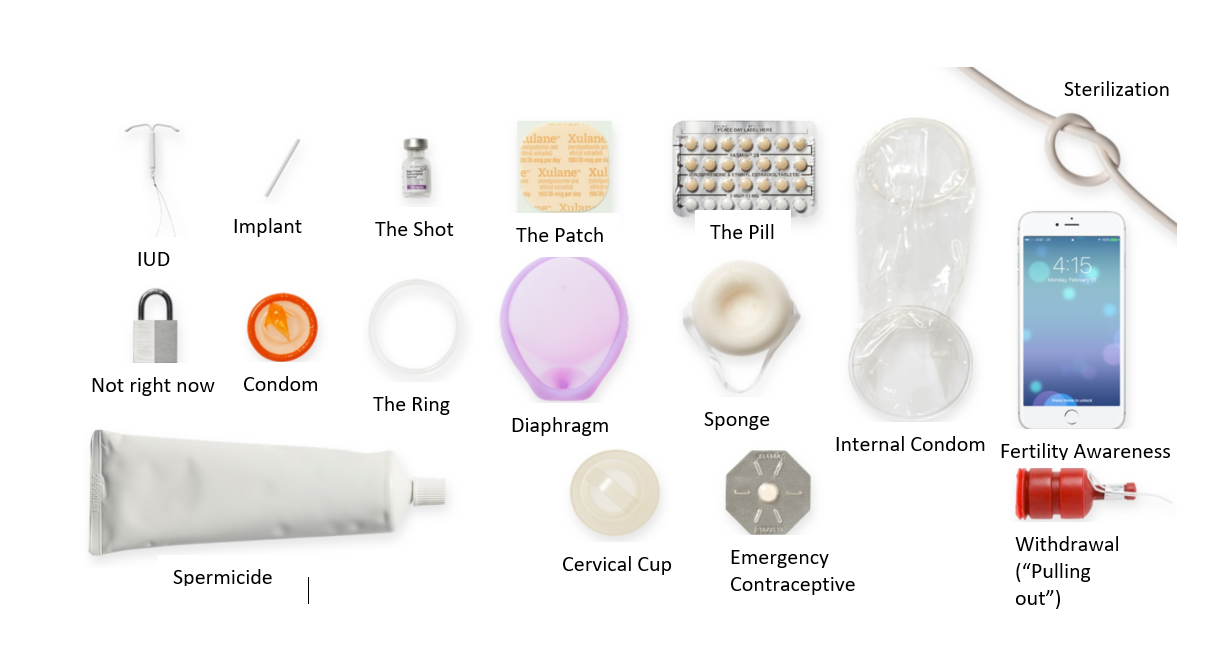


Bedsider.org

1. Which method of birth control have you ever used? [Circle all that apply]


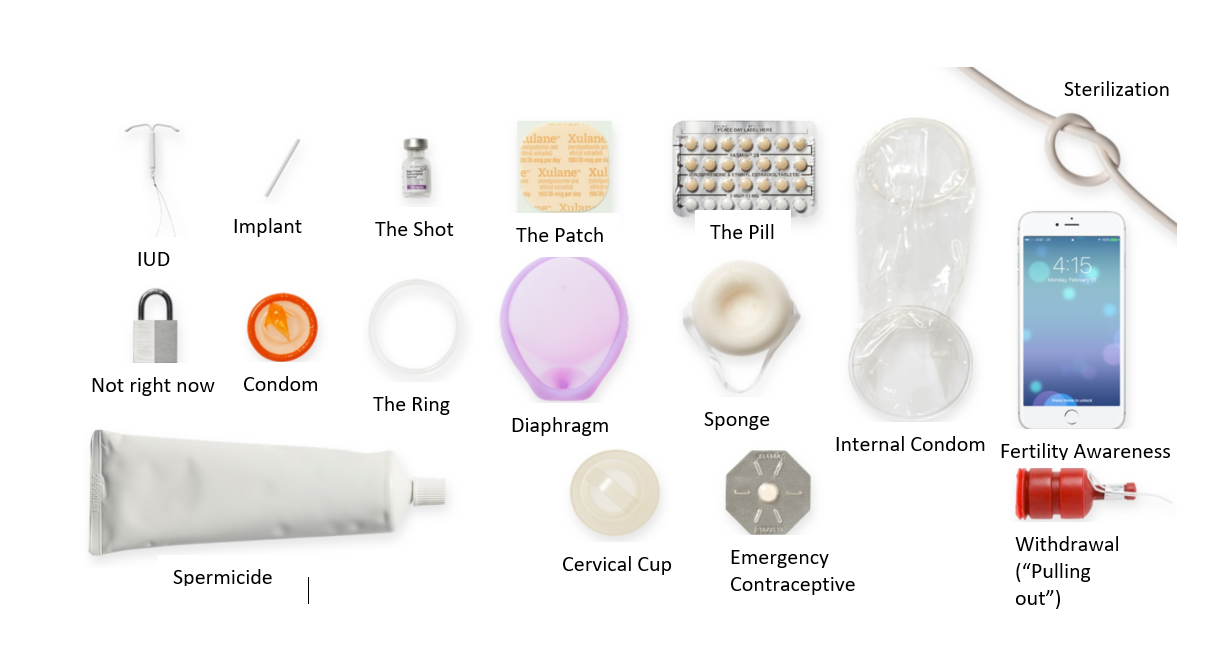


Bedsider.org

6b. How did you decide which method to use? __________________________________________________________________________________________________________________________________________________________________________

6c. Did anyone help you make this decision?

__________________________________________________________________________________________________________________________________________________________________________
