# Supplementary material for: CHOICE-AYA: adapting an evidence-based contraceptive counseling intervention for adolescents and young adults experiencing homelessness
Source: Front Reprod Health. 2026 Apr 10;8:1787996. doi: 10.3389/frph.2026.1787996 (PMC13106319; doi:10.3389/frph.2026.1787996)
Supplement: Supplementary file 2 [file Table2.docx]

**Focus Group Guide**

***Broad exploration of attitudes and opinions on reproductive health care and contraception***

*First, I want to hear about, in general, what teens think about birth control and testing for STDs.*

- What is most important to you when making a choice about birth control?
- What do you think keeps teens from going to get birth control?
  - What barriers or things that have gotten in your way of using a certain method?
  - What are some things you have heard that may make you not want to use a certain method?
- What do you think keeps teens from going to get an STD test?
  - What are some of the barriers or things that have gotten in your way of getting an STD test?

***Prior experiences with healthcare providers***

*Now I want to hear your thoughts about talking with healthcare providers about birth control.*

- What makes the difference between a good conversation and a not-so-good conversation about birth control with a healthcare provider?
- Tell me about any past experiences you’ve had talking to health care providers about birth control.
  - What did you like?
  - What did you not like?
  - What do you wish the health care worker did differently?
- What could health care clinics do to make teens feel more comfortable talking about birth control?
- How would you want to be approached about birth control?
  - How would you want to be made aware that birth control is available while in a shelter or in transition between placements?
- Do you want to know what the provider recommends for you, or would you rather think on your own?
- When and how often should the conversation about birth control come up when seeing your provider?
  - Who should bring up the conversation about birth control first?
- How does the provider characteristics, such as gender, race, age, etc., affect your experience when talking about birth control?
  - How much does it matter that the provider is the same gender as you?
  - What about the provider being the same perceived race as you?
  - How does it change your experience when the provider is from the same background as you?

***Prior experience learning about reproductive health care and contraception***

*We’ve heard that many teens learn about birth control and STIs from school, family, and friends…*

- Is this your experience as well?
- How do conversations with family and friends influence your choice in birth control?
  - Does learning about birth control from people in your life present any barriers to getting your desired method of birth control (whether that be preconceived notions, fear, misinformation or otherwise)?
- What, if any, are advantages to learning about birth control from those close to you?
- What, if any, are disadvantages to learning about birth control from those close to you?
- How might conversations with parents and family about birth control, STIs, and sex be improved?
- How do you think your thoughts and opinions about contraceptives would be different if you did not have these conservations with family or friends?
- Previous groups shared they experienced “scare tactics”. Has anyone used “scare tactics” when talking to you about contraceptives, sex, and STIs?
- How could conversations go better when discussing the risks of having sex?
  - What are specific alternatives to scare tactics that might be more effective at presenting risks to teens?
- How could adults reframe the risks in a way that teens respond well to?
  - How would this information stick best?

***Education for partners without pregnancy potential***

*From previous focus groups, we have learned that males often desire more information about birth control methods both for themselves and for female partners, but this topic is rarely addressed with healthcare providers…*

- How do you think providers should bring up the topic of birth control with male patients?
  - When should they talk about birth control with male patients?
  - Should they bring it up at every visit, or only if the male indicates he is interested?
  - How could a provider best ask permission of a male patient to talk about birth control?
- What kind of things do males want to know about birth control?
  - Where do you feel there are knowledge gaps that could be addressed by healthcare providers?
  - What kind of facts about birth control methods usually utilized by female patients (ex. Pills, LARCs) is it important that males are aware of?
- How do you want your partner to talk with you about birth control options?
  - What information do you wish they knew?
- What are good examples of talking with a romantic partner about birth control? What are bad examples?

***Contraceptive counseling script presentation***

*Now I want you to listen to a couple of ways healthcare providers could talk to a teen about birth control (i.e., comparing efficacy first CHOICE script and patient-centered counseling script).*

- What did you think of the way the healthcare provider talked to the patient in each scenario?
  - How easy or hard is it to understand the messaging?
  - How confident would you be in making a decision about birth control with this messaging?
  - How comfortable do you feel with the counseling approach?
  - How would you change the messaging to meet your needs?
- In one counseling approach, the provider presented birth control options presented in order of efficacy—meaning those that are the best at preventing pregnancy are talked about first. What are your thoughts about this approach?
  - Some people worry that focusing on efficacy, or how well a birth control method works, is not good for all women or teens and may make them feel pressured to take birth control that they don’t really want. What are your thoughts about that?
- Do you want to hear about both hormonal and non-hormonal birth control? All options or just some?
- For partners without pregnancy potential, are you interested in hearing this level of detail?

*Let’s go through and revise the script that you all prefer. Now, let’s go through the revised version.*

- How does it sound to you?
- Are there any other suggestions?

***Use of visual aids in reproductive healthcare***

*We’ve heard visual aids are important…*

- What do you think about the use of visual aids in explaining birth control methods?
  - Would you like the option of seeing a visual aid?
  - When would you like the visual aid presented?
  - What kind of things are important to include in a visual aid?
- How do you feel about seeing pictures of the different methods?
  - Do you feel seeing pictures increases your knowledge of the methods?
- How do you feel about seeing the actual devices, for example a provider showing you what an actual intrauterine device looks like?
