# Supplementary material for: CHOICE-AYA: adapting an evidence-based contraceptive counseling intervention for adolescents and young adults experiencing homelessness
Source: Front Reprod Health. 2026 Apr 10;8:1787996. doi: 10.3389/frph.2026.1787996 (PMC13106319; doi:10.3389/frph.2026.1787996)
Supplement: Supplementary file 3 [file Table3.docx]

**Example Patient Centered Contraceptive Counseling Script^1^**

I see you are here today to discuss contraception. Let’s start by telling me what methods you are looking for in a method (for example, pregnancy prevention, period control, acne, etc). I’d also like to hear what you have tried or heard about in the past and what you may be interested in learning more about today.

When you tried x/y/z, what did you like about it? What did you NOT like about it?

- Was there anything that made this hard to obtain or stick to?

So, it sounds like you want a method that... (*Reflect their experience and identify priorities. Examples below*)

...is highly effective at preventing pregnancy and does not lead to heavy or irregular bleeding

...takes away your period and prevents pregnancy

...does not cause wait gain and is easy to take

...does not require you to go to the pharmacy every month

...is private and low/no cost

Before we get into options, let’s check in about your **medical history**….

Now, that I have a better sense of what’s safe for you, can I tell you about some birth control options?

**I have some visuals for us to look at together as we talk…**

***Note: Options below will be presented in an order based on patient identified priorities. Available options may change over time as new contraceptive formulations are approved or access to existing options changes. Update accordingly.*

Pills must be taken everyday around the same time of the day and are about 92% effective with typical use. Common side effects include nausea and irregular bleeding during the first 3 months. Your periods may become shorter and lighter over time and cramping often gets better. There are prescription pills and even one OTC now.

The patch is used weekly and is a small, square shaped sticker that goes on your low hip, low back, upper shoulder or upper back (*use hand to point to location*). The patch is changed every week on the same day. You will wear a patch for three weeks and then on the fourth week, you won’t wear a patch. This is when you would expect your period. You then keep repeating the process, 3 weeks on, 1 week off. Common side effects include some itching or irritation underneath the patch. Irregular bleeding is common during the first three months, but typically gets better with time. Like pills, periods may become shorter and lighter and cramping may get better.

The ring is used monthly and is a small plastic ring that gets inserted into your vagina. It's similar to inserting a tampon. If you insert it and it feels comfortable, then it's in the right spot. You keep the ring in for 3 weeks at a time and then take the ring out for 1 week to have your period. You can also keep the ring in for 4 weeks at a time. With continuous ring use (changing every 4 weeks), you may not have a period at all which is okay when using birth control. Common side effects including some vaginal discharge and irregular bleeding during the first 3 months of use that typically gets better with time. Like the pills and patch, periods may get shorter and lighter and cramping may get better.

Depo-Provera is a shot in the arm that lasts 3 months. You come to clinic to get the injection and don’t have to remember anything other than coming back to clinic in 3 months for your next shot. It’s about 96% effective with typical use in preventing pregnancy. Irregular bleeding is common during the first 3 months, but typically gets better with time. Some women who use Depo Provera for a year don’t have any bleeding at all and that is okay with this method. Depo Provera can increase your appetite and some women gain weight so it’s important to choose healthy food and regular physical activity.

The implant and the IUD are longer-term options that are >99% effective at preventing pregnancy. They are the most effective options we have. The implant goes in your upper arm and lasts for 3 years. Common side effects include irregular bleeding or spotting. This can come and go throughout the time you have the implant. About ¼ of women have heavier bleeding than they did before and about ¼ don’t have any bleeding at all. It is impossible to predict what your response will be.

The IUD goes inside the uterus. There are several options, but the one we most commonly use provides pregnancy prevention for 8 years. Common side effects including irregular bleeding for the first 3-6 months that typically gets better with time. Most women with the IUD may have shorter, lighter periods or no periods at all. If you are interested in the IUD, we can start another method today and get you an appointment to get the IUD placed at our Broadway clinic.

That’s a lot of information. I want to make sure you know that no decision you make today is permanent. All of these options are reversible and do not affect your future fertility.

What methods **sound most interesting**, or do you want to **know more about**? Do you want to hear my **recommendations**?

Great!...*Launch into method specific education*

I also provide every patient with a prescription for **emergency contraception** just in case. You can keep it on hand to use if you have unprotected sex (for example, no condom or condom breaks) in the future.

References

1. Curtis KM. US selected practice recommendations for contraceptive use, 2024. *MMWR Recommendations and Reports*. 2024;73
